# Erdheim–Chester disease: An elusive diagnosis in a 50‐year‐old Ethiopian man presenting with diffuse sclerotic bone lesion

**DOI:** 10.1002/ccr3.9447

**Published:** 2024-09-18

**Authors:** Semir Abdi Usmael, Addisu Alemu Gebrehiywot, Ashenafi Lemma Bekele, Solomon Bishaw Yezengaw, Tekalign Tsegaye Tefera, Hunduma Bikila Bote, Kalkidan Abera Shibeshi, Anteneh Belachew Fantaye

**Affiliations:** ^1^ Department of Internal Medicine Haramaya University College of Health and Medical Science Harar Ethiopia; ^2^ Department of Pathology Haramaya University College of Health and Medical Science Harar Ethiopia; ^3^ Department of Radiology Haramaya University College of Health and Medical Science Harar Ethiopia; ^4^ Department of Radiology Jugel General Hospital Harar Ethiopia; ^5^ Department of Orthopedic and Trauma Surgery Haramaya University College of Health and Medical Science Harar Ethiopia; ^6^ Department of Radiology Harar General Hospital Harar Ethiopia

**Keywords:** case report, Erdheim–Chester disease, Ethiopia, non‐Langerhans cell histiocytosis, Osteosclerosis

## Abstract

**Key Clinical Message:**

Diagnosis of Erdheim–Chester disease (ECD) requires the clinician to be familiar with its various manifestations, classic radiologic and histologic features. This case highlights the significance of considering ECD in any patient presenting with bone pain and symmetric osteosclerosis of long bones of extremities to allow for early diagnosis and treatment.

**Abstract:**

Erdheim–Chester disease (ECD) is a rare non‐Langerhans histiocytic disorder with diverse clinical manifestations, ranging from indolent, localized presentation to life‐threatening, multi‐systemic disease. Delayed or erroneous diagnosis is common. The presence of classic radiographic finding along with foamy histiocytes that is positive for CD68 but negative for CD1a on histologic examination establishes the diagnosis. We report a second case of ECD from Ethiopia. A 50‐year‐old Ethiopian man presented with a 13‐year history of bilateral lower leg bone pain, cold intolerance, somnolence, constipation, impotence, decreased libido, and secondary infertility. The diagnosis was suspected when skeletal X‐ray revealed bilateral symmetric sclerosis of metadiaphysis of femur, tibia, and humerus. The demonstration of foamy histiocytes that were positive for CD68 but negative for CD1a on histologic examination with immunohistochemical staining confirmed the diagnosis. Evaluation for the extent of the disease revealed coated aorta sign, hairy kidney sign, and cystic lesion with ground glass opacity of lung, primary hypothyroidism, and hypergonadotropic hypogonadism. ECD is rare histiocytic neoplasm with wide range of clinical features which often delay the diagnosis. Clinician should be mindful of the various presentations and the classic radiographic and histologic features of ECD. This case highlights the significance of entertaining ECD in any patient presenting with lower leg bone pain and symmetric osteosclerosis of long bones of lower extremities to allow for early diagnosis and treatment.

## 1 INTRODUCTION

Erdheim–Chester disease (ECD) is a rare non‐Langerhans histiocytic disorder with wide range of clinical manifestations. The exact incidence of ECD is unknown. However, around 1000 cases have been reported in the literature^1^, ^2^ and only one case has been reported from Ethiopia.^3^ It primarily affects adult males in their fifth to seventh decade of life.^4^, ^5^

ECD is a clonal neoplastic disorder, marked by hyperactivating mutation of BRAF and/or other component of mitogen‐activated protein kinase (MAPK) signaling pathways which results in clonal proliferation of myeloid progenitor cells and creates chronic uncontrolled inflammation which is primary mediator of organ dysfunction.^6^, ^7^, ^8^

The clinical manifestations of ECD range from asymptomatic disease detected incidentally on imaging to life‐threatening multi‐systemic disease involving the bone, retroperitoneal organs, central nervous system, respiratory system, cardiovascular system, and skin.^9^, ^10^ A defining or pathognomonic feature of ECD is symmetric metadiaphyseal sclerosis of long bones on plain radiographs, PET‐CT, or bone scintigraphy.^11^ Peri‐aortic soft tissue thickening (“coated aorta sign”) and perinephric tissue thickening (“hairy kidney sign”) are additional imaging findings suggestive of ECD.^11^ The presence of lipid laden or foamy histiocytes surrounded by fibrosis that are reactive for CD68 but negative for CD1a on histologic examination confirms the diagnosis of ECD.^12^

Diagnosis of ECD is usually difficult, due to its rarity and varied clinical features. It should be suspected in any patient presenting with lower leg bone pain and diffuse osteosclerosis of long bones of lower extremities. Diagnoses require the presence of characteristics histopathologic features in the proper clinical and radiologic contexts.

We report a second case of ECD from Ethiopia in a 50‐year‐old man presenting with chronic lower extremity pain and diffuse sclerotic lesions of long bone of legs. This case also emphasizes therapeutic challenges in resource‐limited settings.

## 2 CASE HISTORY

A 50‐year‐old male driver from Ethiopia presented with a 13‐year history of bilateral leg pain. The pain was dull aching, felt at distal part of the thigh and proximal part of lower leg bilaterally. It is worsened by movement and relieved by anti‐pain. The pain is mild and intermittent at first but overtime it became severe and persistent disrupting his daily activities and lead to analgesic dependency. During this period, he visited multiple health facilities, but received no definitive diagnosis and treatment. Associated with this, he had a history of cold intolerance, somnolence, poor concentration, excessive fatigue, and constipation. He also gave a history of impotence and decreased libido. His past medical history includes childhood onset bilateral deafness treated with hearing aid device. He is married with two children, but unable to have additional child despite years of trying. Otherwise, he denied any cough, shortness of breath, chest pain, palpitation, orthopnea, body swelling, headache, blurry vision, body weakness, dizziness, abnormal body movement, gait disturbance, polyuria and polydipsia, joint pain and swelling, skin rash, fever, night sweat, weight loss, and other systemic symptom. He did not smoke tobacco, use illicit drugs, or drink alcohol. He has no family history of hereditary skeletal disease or malignancy.

On examination, his vital signs were with in normal range. There was slight tenderness on palpation of distal thigh and proximal tibia. The rest of physical examination was unremarkable.

## 3 METHODS

### 3.1 Investigation

Blood test revealed mild anemia (hemoglobin 12.4 g/dL) and raised erythrocyte sedimentation rate (ESR 62 mm/h). Peripheral morphology reported normocytic normochromic anemia, adequate white blood cell and platelet count without blast or malignant cells. Otherwise, serum electrolyte, renal function test, liver enzymes, serum albumin, and tumor markers were normal (Table 1).

**TABLE 1:** Summary of investigation at initial evaluation.

Variables	Reference range	6/4/2023
White cell count (per μL)	4000–10,000	7600
Hemoglobin (mg/dL)	11–16.5	12.4
MCV (fL)	80–99	81
MCH (pg)	26.5–33.5	28
Platelet count (per μL)	100–300	406
ESR (mm/h)	0–20	62
Chemistry		
AST (IU/L)	12–38	30
ALT (IU/L)	7–42	29
ALP (IU/L)	60–306	300
Creatinine (mg/dL)	0.5–1.2	0.9
Blood urea nitrogen (mg/dL)	7–20	27
Serum protein (mg/dL)	6.5–8.5	6.8
Serum albumin (mg/dL)	3.5–5.5	4.2
Sodium (mmol/L)	136–146	142
Potassium (mmol/L)	3.5–5	3.9
Chloride (mmol/L)	98–107	98
Ionized calcium (mmol/L)	1.1–1.3	1.3
Phosphorus (mg/dL)	4.5–5.5	4.8
Vitamin D (ng/mL)	30–100	100
Intact PTH (pg/mL)	15–68.3	46.1
Hormone analysis		
HsTSH (μIU/mL)	0.35–4.94	6.74
Free T4 (ng/dL)	5–13	4.94
LH (mIU/mL)	0.57–12.07	22.9
FSH (mIU/mL)	0.95–11.95	6.52
Free testosterone (ng/mL)	4.41–35.38	3.39
Prolactin (ng/mL)	2.52–26.81	26.9
Tumor marker PSA (ng/mL)	1–4	1.2
Urinalysis		
Specific gravity		1.010
pH		6.6
Albumin		Negative
Blood		Negative
WBC		2–3
RBC		Negative
Cast		Negative
Genetic test		
BRAF‐V600E mutation		Not available

Abbreviations: ALP, alkaline phosphatase; ALT, Alanine transaminase; AST, Aspartate transaminase; ESR, Erythrocyte sedimentation rate; FSH, Follicular‐stimulating hormone; HsTSH, High‐sensitivity thyroid‐stimulating hormone; Human immunodeficiency virus; LH, Luteinizing hormone; N/A, not available; PSA, Prostate‐specific antigen; PTH, Parathyroid hormone; WBC, white blood cell.

**FIGURE 1:**
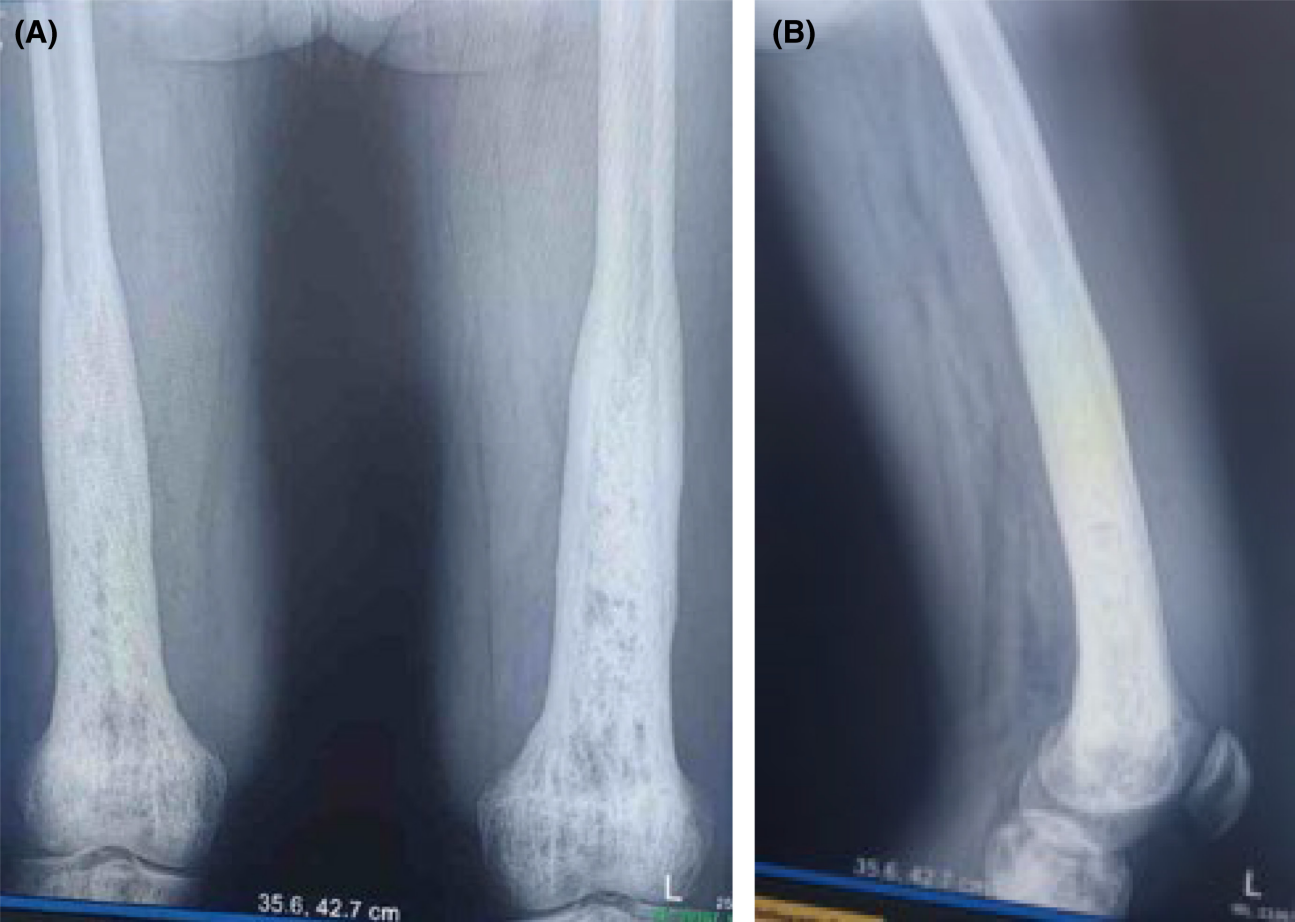
Frontal (A) and lateral (B) radiography of the femur shows bilateral symmetric distal metadiaphyseal cortical thickening and diffuse medullary sclerosis with loss of corticomedullary differentiation.

Skeletal radiography of the femur showed bilateral symmetric distal metadiaphyseal bone expansion with diffuse medullary sclerosis with blurring of corticomedullary differentiation and associated cortical thickening (Figure 1A,B). Similar changes were identified on tibial and humeral X‐ray, but skull, pelvic, and spinal X‐ray were normal. Following a negative metabolic and hematologic work‐up for such sclerotic bone lesions, diagnostic skeletal biopsy was obtained with a consideration of ECD.

**FIGURE 2:**
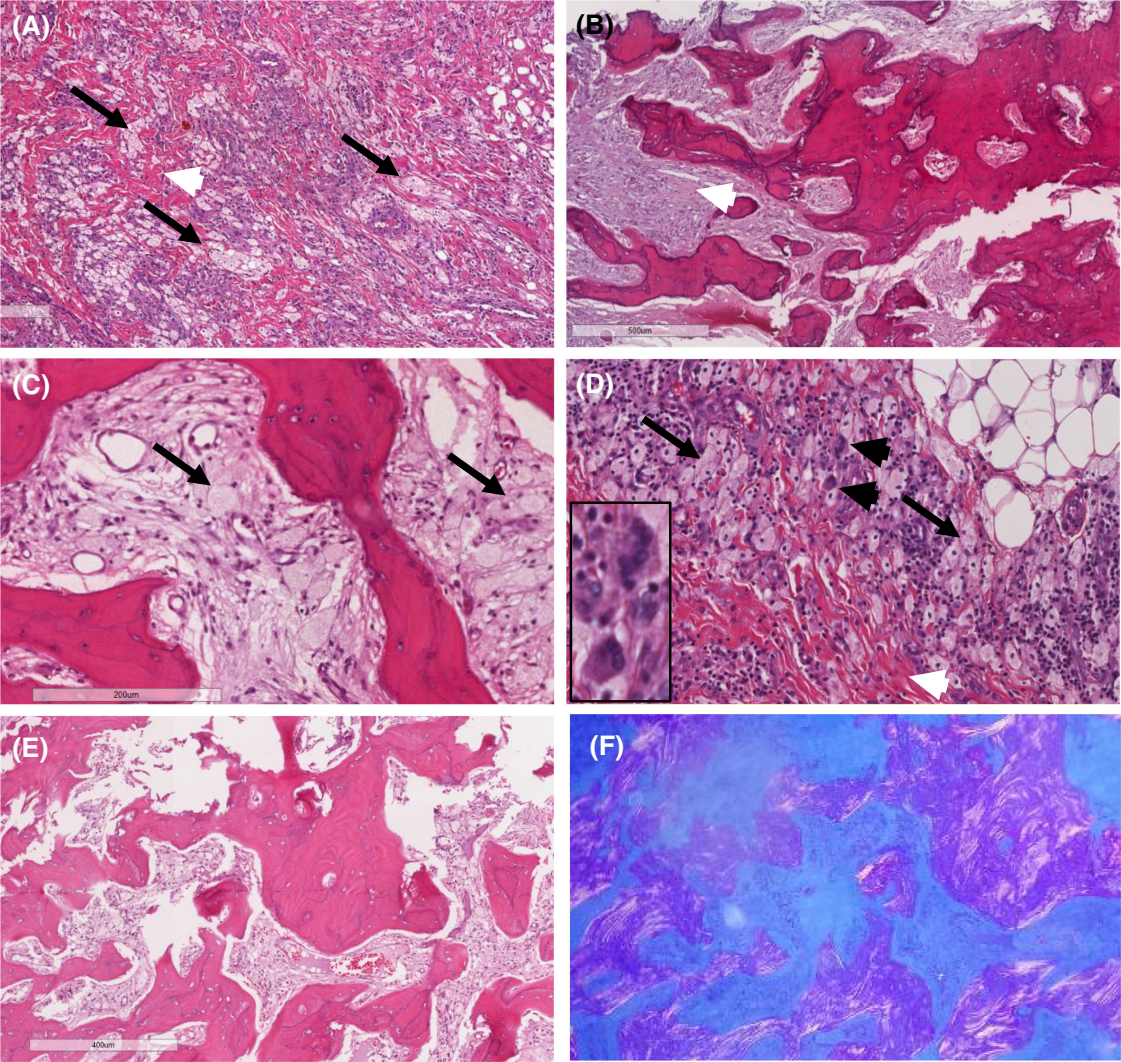
Fibrocollagenous stroma (white arrow head) with clusters of foamy histiocytes (arrow) (A); thickened bony trabeculae with features of cementum surrounding intertrabecular space filled with fibrous stroma (white arrow head) (B) with variable proportion of foamy histiocytes (C). There is also infiltration of the foamy histiocytes accompanied by scattered lymphocytes within the soft tissue (adipose tissue at the right top corner) and rare multinucleated cells (black arrow head and inset) (D). Thickened trabeculae of laminar bone (E) and its character under polarized light (F).

Histopathologic examination of bone and fibro‐adipose tissue demonstrated infiltration with foamy histiocytes admixed with scattered spindle cells, and occasional multi‐nucleated giant cell (Touton cells) and surrounded by fibrosis along with focal cholesterol cleft of xanthogranulomatous reaction (Figure 2A–F). On immunohistochemical staining, the histiocytes were strongly reactive for CD68 and dimly reactive for S100 but negative for CD1a.

After confirming the diagnosis of ECD, several laboratory and imaging tests were done to determine the extent of the disease. Laboratory values were compatible with primary hypothyroidism (high sensitivity TSH 6.74 μIU/mL, Free T4 4.94 ng/dL), hypergonadotropic hypogonadism (LH 22.9 μIU/mL, FSH 6.52 μIU/mL, free testosterone 3.39 ng/mL) (Table 1).

**FIGURE 3:**
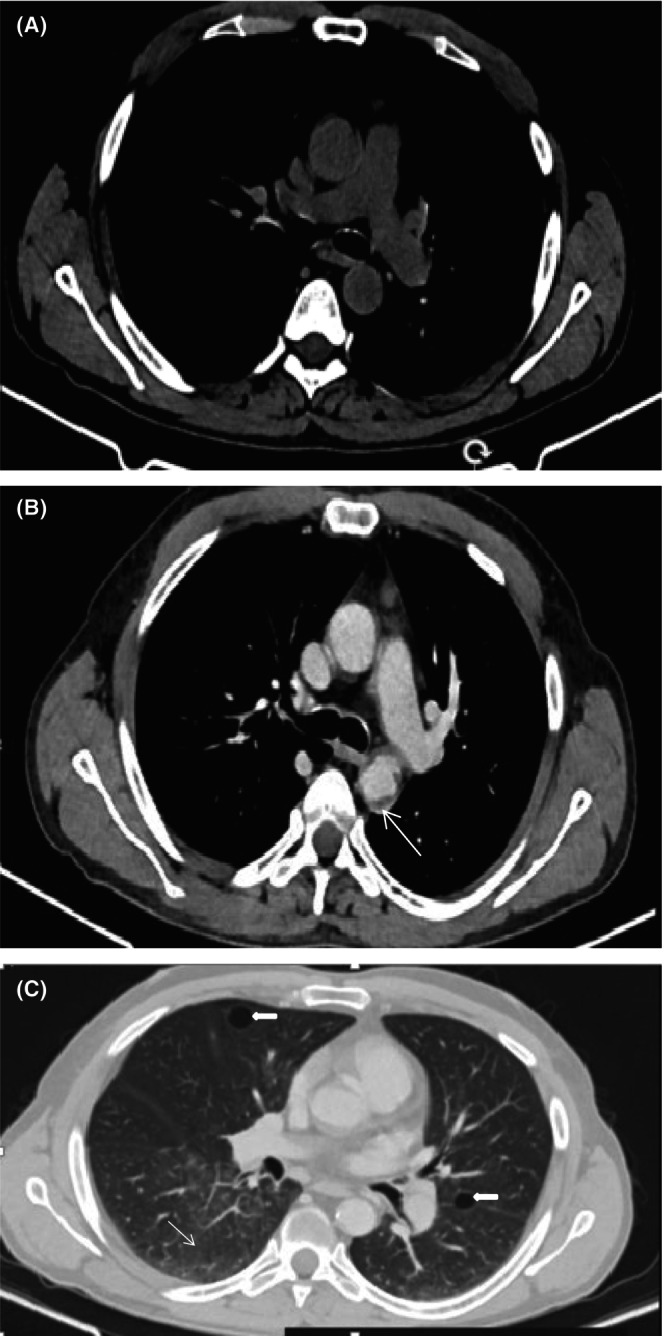
Axial pre‐contrast (A) and post‐contrast (B) chest CT shows soft‐tissue encasement of the aortic arch and descending thoracic aorta (coated aorta sign) (arrow). On lung window (C), there are randomly distributed multiple different sized lung cysts (thick arrow) with right lower lung ground glass opacities (thin arrow).

**FIGURE 4:**
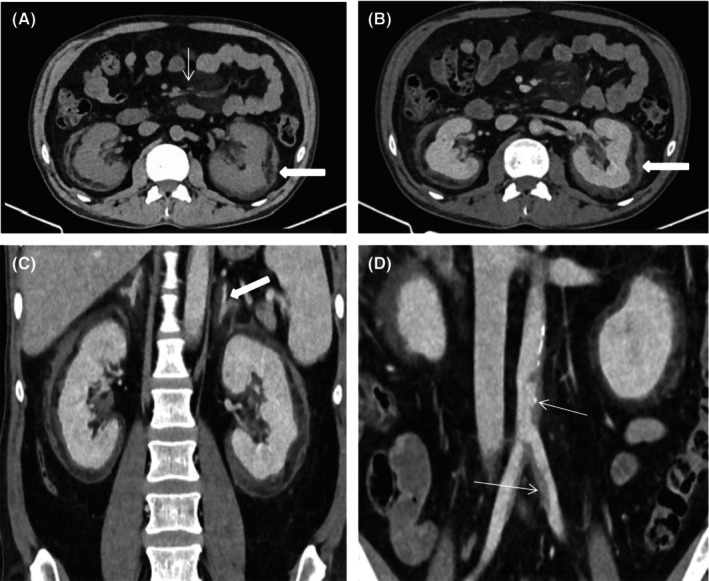
Axial (A and B) contrast‐enhanced CT of abdomen shows ill‐defined, plaque‐like, heterogeneous soft tissue lesion infiltrating bilateral peri‐renal spaces with thickening of anterior and posterior renal fascia (hairy kidney sign; thick arrow) and no sign of hydronephrosis is seen. On the same images, there is fat stranding of the mesentery (thin arrow). Coronal view (C and D) shows mild soft tissue encasement of the abdominal aorta and the proximal bilateral common iliac arteries (Coated aorta sign) (thin arrow) and bilateral adrenal glands (thick arrow).

Contrast‐enhanced chest CT detected mild soft tissue encasement of aortic arch and thoracic aorta (coated aorta sign) and randomly distributed multiple different size lung cysts with right lower lung zone ground glass opacity (Figure 3A–C). Abdominal CT scan revealed bilateral symmetrical peri‐renal soft tissue infiltration and enhancement (hairy kidney sign) and mild soft tissue encasement of the abdominal aorta and the proximal bilateral common iliac arteries (Figure 4A–D). Thyroid and testicular ultrasound were none revealing. Echocardiography and electrocardiogram were unremarkable. Genetic test for BRAF mutation was not determined due to financial reason.

### 3.2 Diagnosis and differential diagnosis

With above evidence, a diagnosis of multi‐systemic ECD involving bone, retroperitoneum, lung, thyroid gland, and testis was established.

ECD must be distinguished from other histiocytic disorders such Langerhans cell histiocytosis (LCH) and Rosai–Dorfman disease (RDD). Both ECD and LCH involve multiple sites, most commonly bones. Localization of sclerotic lesion to distal ends of limbs, absence of birbeck granules, and nuclear grooves along with CD68 reactivity makes LCH unlikely. RDD is histologically distinguished from ECD because macrophages have normal appearing lymphocyte residing in the macrophage cytoplasm. The sclerotic lesions of bone in ECD should be distinguished from variety of metabolic bone disorders such as Paget's disease and POEMS syndrome. However, radiographic, histologic, and immunophenotypic findings make this group of disorders unlikely in this patient.

## 4 RESULTS

He was treated with oral analgesic, levothyroxine 25 μg po daily and subsequently offered treatment with cladribine but he could not afford it. Although we reached out to several foreign organizations, but unfortunately, we could not secure the support we hoped for. The fact that we could not commence appropriate treatment after a decade of agonizing pain and misdiagnosis is disheartening and frustrating both for the patient and the clinicians. This case highlights the challenge in diagnosis and management of such rare disease in a resource‐limited setting such as ours.

## 5 DISCUSSION

Histiocytic neoplasms are rare neoplasms that arise from myeloid lineage cells, namely mononuclear phagocytic cells (macrophage and dendritic cells) or histiocytes. These conditions comprise ECD, LCH, and RDD.^13^

ECD is a rare non‐Langerhans histiocytic disorder. Around 1000 cases have been reported.^4^, ^5^ Similar to our case, it predominantly affects adult males between the fifth and seventh decades of life.

ECD is a clonal neoplastic disorder of unknown etiology. Somatic activating mutations in BRAFV600E and other components of MAPK pathway appear to derive.^6^, ^7^ This activating mutation is found in more than 50% of cases.^14^ Proinflammatory cytokine released by ECD histiocytes causes chronic uncontrolled inflammation and fibrosis, which are the primary mediators of organ dysfunction.^15^

ECD has a wide range of presentations that varies from indolent, localized asymptomatic disease to rapidly progressive life‐threatening multi‐systemic disease. The clinical feature varies depending on the organ involved, the most commonly affected tissue includes the skeleton, vascular, retroperitoneum, endocrine, cardiac, pulmonary, central nervous system, and orbit.

Radiographic skeletal lesion is seen in 95% of cases; however, only 50% of patients experience bone pain as their initial symptom. ECD is characterized by bilateral symmetric sclerosis of the metadiaphysis of the long bones.^11^ Long bones of lower extremities are most commonly involved.^16^ Sclerotic lesion of the long bone of upper extremities and skull particularly facial bones has also been described.^16^ Unlike ECD which typically affect distal end of limbs, LCH most commonly involve the skull, pelvis, proximal limb, and scapula.^17^ Our patient presented with a 13‐year history of progressive bilateral distal thigh pain coupled with a classic radiographic lesion involving distal femur, proximal tibia, and distal humerus.

Cardiovascular involvement occurs in majority of patients and is a substantial cause of morbidity and mortality. It is usually discovered incidentally on imaging.^18^, ^19^ The most common abnormality, known as the “coated aorta,” is seen in two‐thirds of patients and it is caused by circumferential soft‐tissue thickening and encasement of the thoracic and abdominal aorta and its branches.^20^, ^21^ In our case, chest and abdominal CT scans revealed asymptomatic soft‐tissue encasement of the aortic arch, thoracic aorta, abdominal aorta, and common iliac arteries.

Retroperitoneal infiltration by histiocytes is a frequent feature of ECD, occurring in 30%–50% of cases.^22^ Most remain asymptomatic for years. Symptoms may include flank or abdominal pain, dysuria, and slowly progressive renal failure.^23^ Diffuse bilateral infiltration of perinephric tissue results in the so‐called “hairy kidney” and may cause hydronephrosis and ureteral obstruction.^24^ In our patient, an abdominal CT scan revealed a bilateral hairy kidney sign without hydronephrosis.

Pulmonary involvement is seen in 25%–50% of ECD patients. It is usually asymptomatic, but dyspnea and cough might occur on rare occasions.^23^ Findings on CT scan include interlobular septal thickening, ground‐glass opacities, centrilobular opacities, or lung cysts.^25^ In our patient, chest CT revealed asymptomatic lung cysts as well as ground glass opacities in right lower lung zone.

Endocrine manifestations are relatively common and any endocrine gland can be involved. The pituitary is the most commonly affected gland and it commonly presents as diabetes insipidus.^26^, ^27^ ECD can also infiltrate any peripheral endocrine gland. Testis is an unusual site of involvement of ECD. Sonographic signs of testicular infiltration might be seen; however, this does not necessarily correspond with testosterone levels or sperm count.^26^ The patient may be asymptomatic or present with infertility, erectile dysfunction, and decreased libido. The laboratory tests point to hypergonadotropic hypogonadism. Our patient had a history of decreased libido, impotence, and secondary infertility. Low serum‐free testosterone combine with an elevated serum LH level suggests hypergonadotropic hypogonadism. Thyroid gland involvement is very rare.^28^ It may manifest as a palpable nodule or goiter. It may result in subclinical or primary hypothyroidism. In our patient, primary hypothyroidism was confirmed by thyroid function test.

ECD is challenging to diagnose due to its rarity and wide range of presentation. The diagnosis of ECD is based on identifying the characteristic histologic features in an appropriate clinical and radiologic context.^23^

Histologic examination of the lesion typically demonstrates lipid‐laden or foamy histiocytes admixed with inflammation and fibrosis.^12^ On IHC staining, histiocytes are positive for CD68, CD163, and occasionally S100 but negative for CD1a and langerin. Unlike ECD, LCH expresses CD1a and langerin.^12^ To guide therapy with BRAF inhibition, mutational analysis for the BRAF V600E mutation should be performed in all patients.^9^, ^10^

Due to the rarity of ECD, there is a scarcity of evidence from randomized controlled trials and prospective therapeutic studies to guide therapy. Patients with asymptomatic non‐vital single organ or minimally symptomatic (bone or cutaneous) disease can be monitored without treatment. Treatment is reserved for patients with symptoms or evidence of vital organ dysfunction or CNS involvement (including asymptomatic cases).^10^, ^23^ Options of therapy include targeted therapy such as BRAF inhibitors (vemurafenib, dabrafenib), MEK inhibitors (Cobimetinib), mTOR inhibitors (sirolimus), other tyrosine kinase inhibitors (imatinib, sorafenib); conventional therapy such as interferon alpha (IFN‐α) and pegylated interferon alpha (PEG‐IFN‐α); anti‐cytokine biologic agent (anakinra, infliximab, toclizumab), and other systemic therapy (cladribine, glucocorticoids, methotrexate). In patients with BRAF mutation, BRAF inhibitor such as vemurafenib or dabrafenib is the recommended first line of treatment due to its dramatic response in all disease sites.^29^, ^30^ In patient without mutation or access to targeted therapy, IFN‐α and PEG‐IFN‐α are the preferred first‐line agents. However, one of the drawbacks of treatment with interferon or targeted agent is the possibility of recurrence after stopping the medication, requiring a longer duration of therapy.^31^ Thus, in patients who are eligible to receive systemic chemotherapy and/or are unable to access or tolerate targeted agents, a short cycle of cladribine is recommended to achieve sustained response.^32^

ECD is incurable and has poor prognosis. Pulmonary fibrosis, renal failure, secondary to retroperitoneal involvement, and heart failure are the most common cause of death.^33^

Our patient presented with classic features of ECD; chronic lower leg pain along with classic bilateral symmetric metadiaphyseal osteosclerosis of the femur and tibia on skeletal X‐ray. The presence of foamy histiocytes combined with fibrosis that are positive for CD68 and negative for CD1a on histologic examination confirmed the diagnosis. The presence of peri‐aortic soft tissue encasement (coated aorta) and perinephric soft tissue thickening (hairy kidney) on imaging further support the diagnosis of ECD. Even though our patient has an indication for treatment, therapy could not be instituted because of cost and lack of access to the above‐mentioned first‐line medications. The psychological impact of not being able to receive appropriate therapy after a decade of agonizing pain without a definitive diagnosis is immense.

This case highlights many of the diagnostic and therapeutic challenges a clinician from resource‐limited setting faces while caring for patients with rare diseases such as ECD. Establishing the diagnosis is challenging because of lack of expertise, lack of capacity to undertake and evaluate biopsy with IHC staining, lack of ancillary investigations such as mutational tests, and lack of advanced imaging modality. Likewise, managing ECD is also challenging due to the lack of access to first‐line therapeutic drugs and the lack of academic medical centers with expertise in treating ECD. International collaboration and assistance by providing training for clinicians, building the capacity of health facilities, and facilitating access to first‐line medications and inclusion in clinical trials are vital to improve the care and outcome of ECD patients from resource‐limited settings.

There were several limitations in the management of this case namely, the absence of appropriate treatment, lack of brain imaging to rule out asymptomatic CNS lesions, lack of mutational test, and lack of testicular and thyroid biopsy to detect infiltration of these organs.

## 6 CONCLUSION

ECD is a rare histiocytic neoplasm with a wide range of clinical manifestations, posing significant diagnostic and therapeutic challenges. This case highlights the significance of entertaining ECD in any patient presenting with bone pain and diffuse symmetric osteosclerosis of long bones to allow for early diagnosis and treatment. This case also emphasizes the importance of international collaboration and assistance to improve the care and outcome of ECD patients in resource‐limited settings.

## AUTHOR CONTRIBUTIONS

**Semir Abdi Usmael:** Conceptualization; data curation; formal analysis; investigation; methodology; software; supervision; validation; visualization; writing – original draft; writing – review and editing. **Addisu Alemu Gebrehiywot:** Data curation; methodology; resources; software; supervision; validation; visualization; writing – review and editing. **Ashenafi Lemma Bekele:** Data curation; formal analysis; investigation; methodology; project administration; resources; software; visualization; writing – review and editing. **Solomon Bishaw Yezengaw:** Data curation; formal analysis; investigation; methodology; resources; software; validation; visualization; writing – review and editing. **Tekalign Tsegaye Tefera:** Conceptualization; data curation; methodology; software; validation; visualization; writing – review and editing. **Hunduma Bikila Bote:** Data curation; investigation; methodology; software; supervision; validation; visualization; writing – review and editing. **Kalkidan Abera Shibeshi:** Data curation; investigation; software; visualization; writing – review and editing. **Anteneh Belachew Fantaye:** Data curation; investigation; resources; software; validation.

## FUNDING INFORMATION

None.

## CONFLICT OF INTEREST STATEMENT

The authors declare no conflict of interest.

## CONSENT

Written informed consent was obtained from the patient to publish this report in accordance with the journal's patient consent policy.

## Data Availability

Data sharing is not applicable to this article as no new data were created or analyzed in this study.
